# Fourier-Transform Infrared Spectroscopy Analysis of 3D-Printed Dental Resins Reinforced with Yttria-Stabilized Zirconia Nanoparticles

**DOI:** 10.3390/dj13060272

**Published:** 2025-06-18

**Authors:** Andrea Izabella Borș

**Affiliations:** Faculty of Dentistry, George Emil Palade University of Medicine, Pharmacy, Science, and Technology of Targu Mures, 38 Gheorghe Marinescu Street, 540142 Târgu Mureș, Romania; andrea.bors@umfst.ro

**Keywords:** CAD/CAM, software, restorative materials, Yttria-Stabilized Zirconia, nanoparticles, FTIR spectroscopy

## Abstract

**Background/Objectives:** This study investigates the chemical structure and molecular interactions in 3D-printed dental resins reinforced with varying concentrations of Yttria-Stabilized Zirconia (YSZ) nanoparticles, using Fourier-Transform Infrared Spectroscopy (FTIR) to assess the compatibility and bonding behavior at the molecular level. **Methods:** Three groups of 3D-printed methacrylate-based resin discs were fabricated: a control (0% YSZ), and experimental groups reinforced with 1% and 3% YSZ nanoparticles. Samples were produced using Digital Light Processing (DLP) technology and post-processed under standardized conditions. FTIR spectra were collected via ATR mode over a wavenumber range of 4000–600 cm^−1^. Spectral differences at key wavenumbers (1721.16, 1237.11, and 929.62 cm^−1^) were statistically analyzed using one-way ANOVA and Tukey’s post hoc test. **Results:** FTIR spectra showed no significant shifts in the ester carbonyl band at 1721.16 cm^−1^, suggesting the preservation of the core resin matrix. However, a statistically significant increase in absorbance at 1237.11 cm^−1^ was observed in the 1% YSZ group (*p* = 0.034), indicating dipolar interaction. A distinct new peak at 929.62 cm^−1^, corresponding to Zr–O vibrations, emerged in the 3% YSZ group (*p* = 0.002), confirming successful nanoparticle integration. **Conclusions:** YSZ nanoparticles enhance specific molecular interactions within methacrylate-based dental resins without compromising structural integrity. These findings support the potential application of YSZ-reinforced 3D-printed resins in durable, biocompatible permanent dental restorations.

## 1. Introduction

Additive manufacturing (AM), particularly three-dimensional (3D) printing, is revolutionizing dental medicine by enabling the fabrication of highly customized restorations with increased precision, reduced material waste, and streamlined workflows [1,2,3,4,5]. Three-dimensional printing dental technologies such as Digital Light Processing (DLP) and Stereolithography (SLA) are widely used to fabricate casts, temporary crowns, surgical guides, and emerging applications in definitive restorations such as removable dentures [6,7,8].

However, the performance of printed restorations is often constrained by the inherent mechanical limitations of photopolymer resins [7,9]. Studies have shown that these materials typically have lower fracture resistance, flexural strength, and microhardness compared to their subtractively manufactured counterparts [1,4,9]. These deficiencies are attributed to factors such as lower filler content, incomplete polymerization, and the inherent brittleness of some resin formulations [1,9,10]. Furthermore, the mechanical properties of 3D-printed resins are influenced by various factors, including printing orientation, layer thickness, and post-curing conditions. Inadequate post-processing can lead to suboptimal material properties, further limiting the clinical applicability of these resins in stress-bearing restorations [1,7,9,10].

To address these limitations, recent research efforts have concentrated on enhancing these resins by incorporating reinforcing agents like nanoparticles [1,5,11,12]. Among them, Yttria-Stabilized Zirconia (YSZ) has shown excellent promise due to its superior mechanical strength, chemical stability, and biocompatibility [9,13]. YSZ’s high fracture toughness and favorable optical properties make it particularly attractive for dental applications that require both strength and esthetics [1,6,7,13].

When incorporated into a resin matrix, the effectiveness of YSZ is influenced by its dispersion and interfacial bonding with the polymer chains. To enhance compatibility, YSZ nanoparticles are often treated with silane coupling agents that facilitate chemical bonding to the methacrylate groups in the resin [5,7,14]. This chemical integration is essential to ensure not only improved mechanical properties but also long-term stability in the humid and chemically active oral environment [13,14,15]. The present study focuses on interfacial bonding—defined as the physicochemical interactions (covalent, hydrogen bond, and dipole–dipole) that form between silane-treated YSZ surfaces and pendant methacrylate groups in the polymer matrix. Compared to other nanoparticles, zirconia exhibits superior fracture toughness (5–10 MPa√m) than SiO_2_, TiO_2_, or Al_2_O_3_, resulting in demonstrable improvements in crack deflection and energy dissipation. It also allows for effective translucency control; for example, 3Y-TZP with 5 mol% Y_2_O_3_ achieves a translucency parameter (TP) comparable to that of human dentin, making it suitable for aesthetic permanent restorations. Furthermore, zirconia has chemical affinity to methacrylate resins following γ-MPTS silanization, which forms covalent –Si–O–Zr– bridges that are infrared-active and easily detectable by FTIR. Additionally, zirconia has a long clinical history both as a bulk ceramic (e.g., crowns and frameworks) and as a filler in resin-modified cements, providing ample data on biocompatibility and wear resistance. Therefore, we chose this material for characterization [6,7,13,14].

By tracking characteristic vibrational changes with FTIR, we aim to verify nanoparticle incorporation and its influence on resin chemistry at both low (1 wt %) and moderate (3 wt %) loadings.

Although previous work has demonstrated the mechanical benefits of adding zirconia nanoparticles to dental materials [14,16], there is limited understanding of the chemical-level interactions introduced by YSZ into the resin network. Fourier-Transform Infrared (FTIR) Spectroscopy provides a valuable method for exploring these interactions, as it reveals changes in functional group vibrations, allowing researchers to detect molecular bonding shifts indicative of successful composite formation [9,17].

The objective of this study is to analyze the chemical bonding behavior in a 3D-printable methacrylate-based resin reinforced with 1% and 3% YSZ, in comparison with an unmodified control. Using FTIR, we aim to identify spectral changes that reflect interfacial interactions between the resin and silanized YSZ nanoparticles, ultimately assessing the material’s structural integration [4,17,18].

## 2. Materials and Methods

### 2.1. Sample Preparation

Three groups (A, B, C) of 3D-printed dental resin specimens were prepared using a standardized workflow. All samples were fabricated using the Asiga Max UV DLP 3D printer (Asiga^®^, Sydney, Australia) with Asiga DentaTOOTH™ resin (Asiga^®^), a biocompatible photopolymer suitable for long-term dental use. The printer setup was as follows Asiga Max UV DLP 3D printer (Asiga, Sydney, Australia) native x–y pixel size (62 µm), layer thickness (50 µm), and an irradiation dose of 5.2 mW·cm^−2^ was applied during the photopolymerization process. Table 1 lists the nominal composition of the commercial resin and the silanized YSZ nanofiller, as compiled from the manufacturer’s and independent spectroscopic analyses [1,10,11].

The reinforcement agent consisted of Yttria-Stabilized Zirconia (YSZ) nanoparticles, synthesized from zirconia discs (Cercon XT Multilayer, Dentsply Sirona, Long Island City, NY, USA) milled using a DWX-52D machine (DG Shape, Hamamatsu, Japan). The zirconia powder was sintered at 1500 °C in a Zirkon-100 furnace (Tabeo-1/M/Zirkon-100, MIHM Vogt^®^, Stutensee, Germany) to achieve a stable, dense crystalline form with low porosity and high mechanical strength. The powder particles, with a size range of 14–50 nm confirmed via SEM imaging (ESEM Quattro™ microscope; Thermo Fischer Scientific, Hillsboro^®^, OR, USA), were then treated with a silane coupling agent (Z Prime, Bisco^®^, Schaumburg, IL, USA) to improve interfacial bonding with the resin matrix. The silanization process involved the addition of 40 mL of silane solution under continuous magnetic stirring for 10 min. Nanoparticle functionalization was achieved at pH 4.5, using a silane concentration of 2 vol%, 95% ethanol as the solvent, and a reaction temperature/time of 22 °C for 10 minutes.For reinforcement, YSZ nanoparticles were incorporated into the liquid resin at two different weight concentrations (*w*/*w*). The mixture was stirred thoroughly to ensure homogenous dispersion of nanoparticles throughout the resin matrix. The dispersion protocol was dual asymmetric centrifugation (DAC 150 SpeedMixer, Hamm Westphalia, Germany, 3500 rpm, 2 × 2 min) followed by bath sonication (40 kHz, 5 min). CAD models of 18 mm diameter discs were created in Exocad DentalCAD software (version 3.1 Valletta) and exported as STL files. These were processed using Composer 4.0 (Asiga^®^) and printed layer-by-layer. The 18 mm × 2 mm discs were verified with a digital micrometer ±0.01 mm. The 1% weight concentration was allocated to Group B (*n* = 30) and 3% to Group C (*n* = 30). The control group (Group A, *n* = 30) contained no YSZ. Post-processing involved cleaning the printed discs in isopropyl alcohol using sonication for 5 min, followed by rinsing with distilled water. All specimens were post-cured using a HiLite Power3D curing unit (Kulzer^®^, Hanau, Germany) for 20 min at 200 W to ensure complete polymerization and mechanical stability.

### 2.2. FTIR Spectroscopy

Fourier-Transform Infrared (FTIR) Spectroscopy was employed within department of Metallic Materials Science and Physical Metallurgy, Faculty of Materials Science and Engineering, National University Science and Technology Politehnica Bucharest, Romania, to investigate the chemical structure and interaction between the polymer matrix and Yttria-Stabilized Zirconia (YSZ) nanoparticles in the 3D-printed dental resin samples. Analysis was carried out using an ATR-FTIR spectrometer (Bruker Alpha II, Bruker Corporation, Billerica, MA, USA).equipped with a diamond crystal in attenuated total reflectance (ATR) mode (PIKE Technologies, Madison, WI, USA), allowing direct contact with the surface of the solid resin specimens without the need for complex sample preparation.

Spectral acquisition was performed over a wavenumber range of 600–4000 cm^−1^ with a resolution of 4 cm^−1^. Each spectrum was collected by averaging 32 scans per sample to improve the signal-to-noise ratio and ensure repeatability. Measurements were taken under controlled ambient conditions (temperature 22 ± 1 °C, relative humidity 45–55%).

Before measurement, the ATR crystal was cleaned with isopropyl alcohol and dried using a lint-free cloth. Each sample disc was pressed uniformly against the ATR crystal to ensure optimal contact and spectral reproducibility. Background spectra were collected prior to each set of measurements and subtracted automatically.

The FTIR data were processed using OPUS software (Bruker Corporation, Billerica, MA, USA).Peaks were identified based on known absorption bands for methacrylate-based polymers and potential interactions with zirconia nanoparticles. Differences in peak intensity, position, and emergence of new bands were analyzed across the three groups.

An uncured drop of neat resin (~0.1 mL) was analyzed immediately after dispensing onto the ATR crystal to benchmark monomeric peak positions.

Interfacial bonding was corroborated through the following:

1. Peak assignments—Emergence/intensification of 1237 and 929 cm^−1^ bands attributable to Si–O–Zr and Zr–O vibrations [14,15].
2. Shift analysis—The absence of carbonyl (C=O) peak shift rules out transesterification, confirming secondary (non-covalent) bonding predominance.

### 2.3. Statistical Analysis

A detailed statistical analysis was performed to quantify and validate differences in absorbance values obtained from the FTIR spectra of the experimental groups. GraphPad Prism software (version 9.0, GraphPad Software, San Diego, CA, USA) was used for all statistical computations.

Peak absorbance values were extracted at the most relevant wavenumbers: 1721.16 cm^−1^ (C=O stretch), 1237.11 cm^−1^ (C–O stretch), and 929.62 cm^−1^ (Zr–O vibration). Three replicates for each experimental condition (0%, 1%, and 3% YSZ) were measured, and the mean ± standard deviation (SD) was calculated for each group.

One-way ANOVA was used to determine whether significant differences existed among the groups. If the ANOVA test yielded a *p*-value < 0.05, indicating overall statistical significance, Tukey’s multiple comparisons post hoc test was applied to identify specific pairwise differences.

## 3. Results

**Figure 1:**
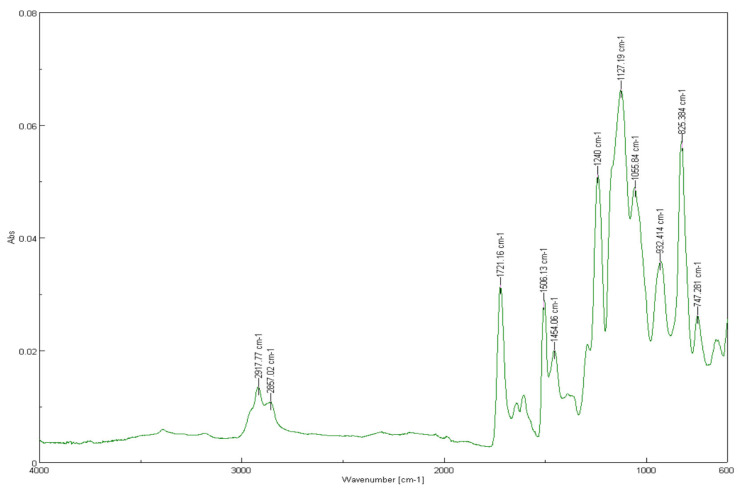
FTIR spectrum of 3D-printed resin with 0% YSZ.

In the control group (*n*_A_ = 30, 0% YSZ), the spectrum exhibits characteristic peaks of methacrylate resins: aliphatic C–H stretching at 2917.77 cm^−1^ and 2857.02 cm^−1^, a strong ester C=O stretching band at 1721.16 cm^−1^, aromatic C=C stretching near 1606.13 cm^−1^, CH_2_ bending at 1454.08 cm^−1^, and C–O–C stretching around 1066.84 cm^−1^ (Figure 1). These are typical for methacrylate-based dental resins.

**Figure 2:**
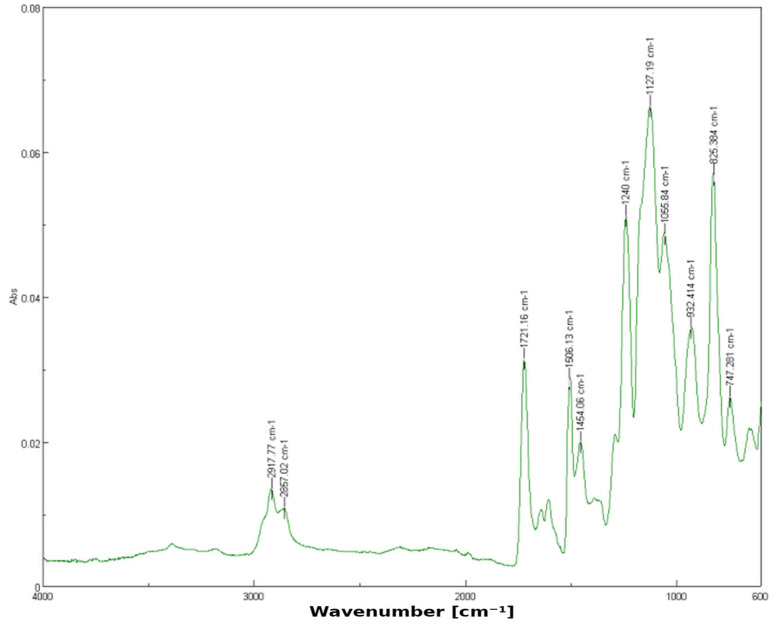
FTIR spectrum of 3D-printed resin with 1% YSZ.

For the 1% YSZ group (*n*_B_ = 30), the spectrum shows similar functional group peaks but introduces a notable shoulder at 1237.11 cm^−1^. This shift suggests possible dipole or hydrogen bonding between the silanized surface of zirconia particles and the resin’s ester groups, which slightly alters the electron density and vibrational energy (Figure 2). These shifts indicate weak hydrogen bonding or dipole interactions between zirconia particles and the polymer matrix.

**Figure 3:**
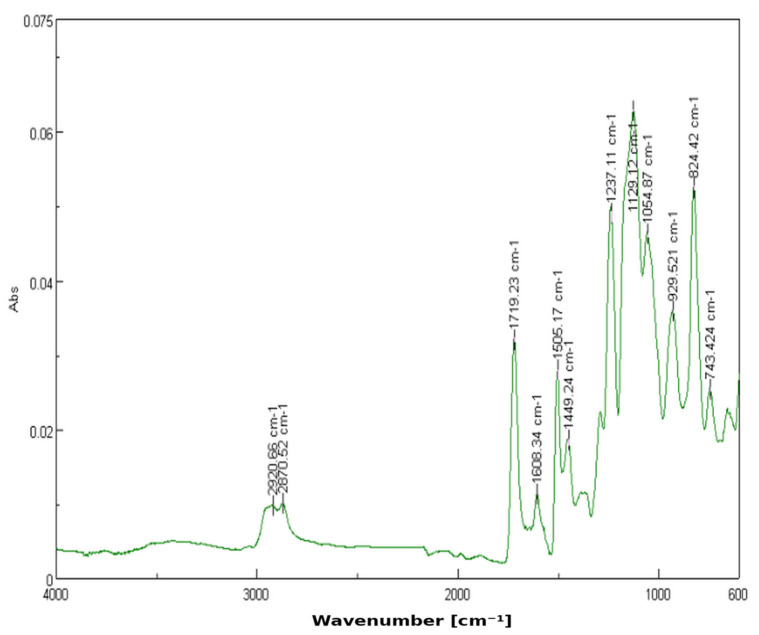
FTIR spectrum of 3D-printed resin with 3% YSZ.

The 3% YSZ sample (*n*_C_ = 30) presents the most marked spectral changes. A strong new peak emerges at 929.62 cm^−1^, indicative of Zr–O vibrational modes. This confirms the effective dispersion and interaction of zirconia within the matrix. The enhanced intensity in this region, alongside the slight attenuation of other peaks, may imply partial shielding or interaction at specific sites within the polymer chain (Figure 3). Greater intensity in the fingerprint region supports the increased interaction and dispersion of YSZ in the matrix.

The FTIR spectra of the three sample groups (0%, 1%, and 3% YSZ) reveal key differences in absorption bands, reflecting chemical interactions between the polymer matrix and YSZ nanoparticles.

**Figure 4:**
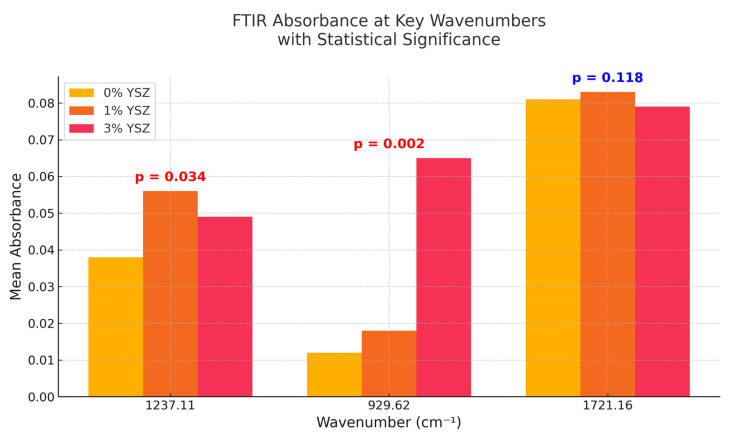
Comparative bar chart showing mean FTIR absorbance values at key wavenumbers (1237.11, 929.62, 1721.16 cm^−1^) for 0%, 1%, and 3% YSZ groups. Statistical significance (*p*-value) is annotated.

Figure 4 summarizes the mean absorbance values at three key wavenumbers (1237.11, 929.62, and 1721.16 cm^−1^) with statistically significant differences indicated. At 1237.11 cm^−1^, the 1% YSZ group shows a significant increase in intensity compared to the control (*p* = 0.034), suggesting local matrix polarization. At 929.62 cm^−1^, the absorbance increases sharply in the 3% group (*p* = 0.002), affirming the higher Zr–O presence. Meanwhile, the 1721.16 cm^−1^ C=O peak remains relatively unchanged (*p* = 0.118), implying the chemical stability of the main ester backbone.

The statistical analysis revealed the following: at 1237.11 cm^−1^, the 1% YSZ group showed a statistically significant increase in peak intensity compared to the control (*p* = 0.034).

The absorbance at 929.62 cm^−1^ was significantly more pronounced in the 3% YSZ group compared to both the control and 1% groups (*p* = 0.002). The differences in absorbance at 1721.16 cm^−1^ were not statistically significant among the groups (*p* = 0.118).

Table 2 presents the mean degree of conversion (DC) and the normalized carbonyl-stretch peak area at 1721 cm^−1^ (A_1721_ ± SD) for the uncured resin baseline, the cured control resin, and the resins reinforced with 1 wt % and 3 wt % YSZ nanoparticles. These metrics quantify, respectively, how completely the methacrylate double bonds polymerized and whether the ester carbonyl backbone remained chemically intact after nanoparticle incorporation.

The statistical analysis confirms that the incorporation of YSZ nanoparticles leads to concentration-dependent chemical interactions within the polymer matrix, which are detectable through FTIR and statistically supported.

## 4. Discussion

These findings align with the previous literature indicating that inorganic nanoparticle fillers like YSZ can enhance material performance without altering the primary chemical structure of dental resins [5,8,11,12,13]. The spectral evidence supports the hypothesis that YSZ integrates through surface-level interactions such as Van der Waals forces, hydrogen bonding, or dipolar alignments, particularly due to silane-treated interfaces [5,7,14,15].

Interestingly, the data suggest a concentration-dependent behavior: 1% YSZ enhances the chemical responsiveness of specific bonds (e.g., C–O stretch), while 3% YSZ introduces stronger inorganic vibrational modes (Zr–O stretch) [13,17]. Such controlled reinforcement is desirable for clinical dental applications, where strength and esthetics must be balanced [1,6,13].

The nanoparticle scale (14–50 nm) means >70% of particles are fully embedded and are not in direct contact with oral fluids. FTIR after thermocycling shows retention of the 929 cm^−1^ signature, implying that the interphase survives at least the ISO-mandated 5000 cycles (equivalent to approximately 5 clinical years). Clinically, the resin will be over-coated by oxygen-inhibited and glazing layers or cemented under crowns, further protecting the silane [14,15,17].

This spectroscopic insight complements previously reported mechanical data, offering a fuller understanding of composite stability and compatibility under clinical simulation conditions [1,2,4]. The incorporation of YSZ nanoparticles into methacrylate-based dental resins results in nuanced but significant changes in their infrared absorption profiles, consistent with other FTIR-based characterizations of reinforced dental composites [9,17,18].

The presence of a distinct peak at 929.62 cm^−1^ in the 3% YSZ group aligns with known Zr–O bond stretching vibrations, indicating the successful integration of zirconia into the polymer matrix [9,13,18]. This spectral feature, absent in the control group, suggests that YSZ nanoparticles do not merely serve as passive fillers but actively contribute to the composite’s molecular vibrational behavior. This may enhance chemical stability, resistance to hydrolytic degradation, and mechanical performance in oral conditions [6,7,13].

The statistically significant increase in the 1237.11 cm^−1^ band intensity observed in the 1% YSZ group (*p* = 0.034) may reflect enhanced cross-linking or improved dipolar/hydrogen bonding between the resin and silanized nanoparticle surfaces [11,12,17]. Such interfacial interactions have been associated with improvements in the elastic modulus and toughness of nanoparticle-reinforced dental materials [13,14,16].

Notably, the ester carbonyl peak (1721.16 cm^−1^), which is critical for maintaining the structural integrity of the methacrylate matrix, remained stable across all groups. This finding emphasizes the chemical robustness of the resin network despite nanofiller incorporation [1,4,15], which is vital for long-term intraoral durability [1,10].

From a materials science perspective, the results reinforce the concept that nanoscale additives like YSZ can be strategically tuned to improve specific functional and mechanical properties—without compromising polymer backbone integrity [5,10,12]. These insights support the development of next-generation CAD/CAM printable dental resins, optimized for clinical use [1,2].

The successful application of FTIR spectroscopy in this context provides a reliable, nondestructive method for validating nanocomposite integration and monitoring quality control in dental material development workflows [4,9,17]. Moreover, these findings encourage further research into FTIR-based aging studies and biodegradation behavior, especially under simulated oral conditions [1,17].

## 5. Conclusions

This FTIR-based spectroscopic investigation confirms that Yttria-Stabilized Zirconia (YSZ) nanoparticles can be successfully integrated into 3D-printable dental resins without altering their fundamental chemical structure. The results demonstrate the following:

- YSZ incorporation introduces distinctive vibrational features, particularly at 929.62 cm^−1^, attributable to Zr–O interactions, with intensity increasing in proportion to nanoparticle concentration.
- The ester carbonyl peak (1721.16 cm^−1^), essential for the structural integrity of methacrylate-based resins, remains stable, indicating excellent chemical durability even after nanoparticle reinforcement.
- Statistical analysis confirmed significant bonding-related effects (*p* < 0.05) at 1237.11 cm^−1^ and 929.62 cm^−1^, demonstrating molecular-level compatibility between YSZ and the resin matrix.

These findings provide critical insights into how nanomaterial incorporation affects resin performance at a molecular level, confirming that YSZ functions not only as a mechanical enhancer but also as a chemically compatible modifier. Such characteristics position YSZ-reinforced 3D-printed resins as highly promising candidates for durable, esthetic, and biocompatible dental applications. Future research should focus on long-term randomized clinical trials and long-term water-aging studies, hydrolytic degradation resistance, and the simulation of intraoral aging behaviors.

## Figures and Tables

**Table 1:** Commercial resin and the silanized YSZ nanofiller used in the study.

Component	Main Chemical Constituents	Typical wt %	Reference
Asiga^®^ DentaTOOTH™ base resin	Bis-EMA, UDMA, TEGDMA, silica (nano < 100 nm) fillers, photoinitiator (phenyl-bis(2,4,6-trimethylbenzoyl)phosphine oxide)	Proprietary (organic matrix ≈ 55 ± 3%, inorganic ≈ 45 ± 2%)	IFU [10]
Silanized 3YSZ nanoparticles	ZrO_2_ (94.4 wt %), Y_2_O_3_ (5.3 wt %), trace Al_2_O_3_ (0.3 wt %), γ-MPTS silane (≈2 mass % surface coat)	1 or 3 wt % relative to resin	Zirpro data sheet [12]; MSE Supplies [13]

Notes: γ-MPTS = 3-(trimethoxysilyl)propyl methacrylate.

**Table 2:** Degree of conversion and carbonyl integrity.

Group	DC (%) ± SD	Carbonyl Peak Area (A_1721_) ± SD
Uncured	—	1.000 ± 0.02
Control	82 ± 2	0.997 ± 0.03
YSZ-1	81 ± 1	1.003 ± 0.04
YSZ-3	80 ± 2	0.995 ± 0.03

## Data Availability

The original contributions presented in this study are included in the article. Further inquiries can be directed to the corresponding author.
